# The Use of a Two-Phase Online Delphi Panel Methodology to Inform the Concurrent Development of a School-Based Ovulatory Menstrual Health Literacy Intervention and Questionnaire

**DOI:** 10.3389/fgwh.2022.826805

**Published:** 2022-05-23

**Authors:** Felicity Roux, Sharyn Burns, HuiJun Chih, Jacqueline Hendriks

**Affiliations:** School of Population Health, Curtin University, Perth, WA, Australia

**Keywords:** Delphi, menstrual cycle, fertility, health literacy, health promoting school model, adolescent girls, mental health, endometriosis

## Abstract

**Introduction:**

There are a high prevalence of ovulatory-menstrual (OM) dysfunction and low levels of menstrual health literacy in adolescents, yet few evidence-based OM health education resources for schools.

**Method:**

This two-phase study used an online Delphi methodology to build consensus across thirty-five purposively selected professionals from the diverse professions of health and education. The panellists were tasked to inform the development of a school-based OM health literacy resources.

**Results:**

In Round One, 86% of panellists determined the scope of these resources using guided and open-ended questions. The study then split into two phases which ran concurrently. In the first phase informing the intervention's development, 57% of panellists participated in Round Two, and 29% reviewed selected lessons. In the second phase informing the questionnaire's development, 51% of panellists participated in Round Two, and 69% in Round Three. The overall consensus reached for the intervention phase and questionnaire phase were 82% and 84%, respectively. The Panel's recommendations included a strengths-based position to counter menstrual stigma, teaching accurate self-report of cycle biomarkers, addressing multiple menstrual dysfunctions and adopting a whole-school approach.

**Conclusion:**

Although time-consuming and requiring a sustained interest, this two-phase Delphi methodology offered anonymity to panellists from distinct professions which facilitated their independent contribution to developing OM health literacy school resources.

## Introduction

Australian studies have observed a high prevalence of menstrual dysfunction in adolescent girls, including over 90% self-reported dysmenorrhoea (1–3) and premenstrual symptoms (1), and up to 40% atypical bleeding patterns (1). Of those adolescents presenting with heavy menstrual bleeding, almost 50% were diagnosed with iron deficiency and/or anaemia (4). Recent studies indicated that young women's menstrual health literacy levels are low, and recommend a review of menstrual health education (5, 6).

The World Health Organisation defines health literacy as “the cognitive and social skills which determine the motivation and ability of individuals to gain access to, understand and use information in ways which promote and maintain health” (7). Schools are important in promoting student well-being (8). The evidence suggests that healthier students have better educational outcomes (9, 10).

However, there are few evidence-based educational programmes that adequately address ovulatory menstrual (OM) health literacy (11). Reasons may include the stigma surrounding menstruation (12, 13) and general lack of awareness around fertility (14–16). Another reason is the lack of preservice training (17) or ongoing professional development for educators in sexual health (18). This lack of skills and confidence may explain schools' reliance on external presenters to deliver menstrual health education (19, 20).

Developing evidence-based educational programmes is demanding. One challenge is reconciling the divergent views of experts in separate professions (21), which in this study were education and health. Whilst education professionals understand the complexity of curriculum development, assessment requirements, and appropriate pedagogy for adolescents, they are unlikely to be experts in the OM cycle. Similarly, whilst healthcare professionals understand the complexity of the OM cycle, its developmental trajectory and pathophysiology, they have little formal training in pedagogy. This tension (22) motivated the use of the Delphi methodology to build consensus among professionals who would not typically engage with each other (23).

Furthermore, there are few instruments available to measure adolescent OM health literacy. As adolescents are developing physically, cognitively and socially towards full adult maturity, their health experiences are particular to their life stage, as are their family and peer dependencies (24). Measurement of their health literacy is therefore different to that of adults (25, 26). Additionally, instruments measuring health literacy differ depending on their use in a health care setting such as a clinic compared to a health promotion setting such as a school (27).

Educationally, health literacy as a learning outcome offers a valuable tool for school-teachers to set learning objectives and to assess their students' achievement (27). The Health Outcome Model (28) is used in schools (25), including the Western Australian School Curriculum and Standards Authority which uses it to set the standards, reporting and assessment of the Health & Physical Education (HPE) curriculum (29). The Model (28) is valued for its empowering approach, whereby mastery of knowledge and skills may help individuals influence the conditions affecting their health experiences (30). It has also been observed that the Model's (28) progression coincides with the trajectory of social and cognitive development in adolescents (31). Health literacy is progressively acquired by sequentially completing and rising through the three domains of the Model's (28) hierarchical structure (26, 32). The first domain of functional health literacy includes basic knowledge and skills related to finding and comprehending health information to function effectively. Once mastered, the second domain is interactive health literacy, which includes more advanced skills related to personally applying health information, communicating with others including healthcare providers, and setting goals to enhance health. Finally, the third domain of critical health literacy includes the most advanced-level skills related to critically analysing health information, applying this to promote own and others' health and improving social awareness.

A protocol to develop and trial a school-based OM health intervention was formulated to address these challenges (33). This article describes the initiation of this protocol with an online two-phase modified Delphi Panel methodology (34) to build consensus between the expertise and perspectives of experts within two distinct professions (35), namely health and education. The Delphi was used to inform the development of the intervention and the questionnaire to measure its impact on OM health literacy. The study's objectives were to obtain content validation (36) that would inform the development of:

1. An OM health literacy intervention for secondary schools (the first phase); and
2. A questionnaire to test adolescent OM health literacy (the second phase).

## Materials and Methods

Accepted practise supports a Delphi Panel's work with pre-existing information, such as from literature reviews (36), which has been recommended for education (37), health-based projects (38) and intervention development (39). This study was supported with information from a systematic literature review of school-based OM health education programmes (11) for the intervention development. The tools used by these reviewed programmes to measure their impact (40–44) informed the study's questionnaire development, including previously validated question items and scales (45–48).

### Participant Recruitment and Engagement

The term “expert” is used loosely in Delphi studies to denote an individual who is knowledgeable, experienced and interested in the subject (34, 36). Experts for this study were recruited purposively from professional networks and snowball technique (23). The selection criteria included formal qualifications (including degrees, professional development certificates or accreditation by peak authorities), specialty practise, publications or active service. Particular care was taken when recruiting fertility awareness instructors. Rigour was maintained by recruiting only those accredited independently by renowned authorities on fertility awareness-based methods. Interested professionals were provided an information statement describing the project's aims, the contribution requested and a consent form. No incentives were offered.

This study included an opt-in opt-out feature (49), whereby panellists could respond to different rounds at their own discretion. This may have reduced the burden of an “all or nothing” participation, particularly given the demands that this methodology can place on panellists' time and ability to sustain interest over several rounds (49).

Delphi methodology prescribes anonymity, hence there were no face-to-face meetings (21, 37, 50–52). Failure to preserve anonymity would have jeopardised this methodology's advantages compared to other group consensus methodologies such as the Nominal Group Technique (NGT). The limitations of NGT include a smaller number of participants and the risk of dominant participants unduly influencing the group (21, 50, 51). The advantages of the Delphi include the flattening of hierarchies and management of the group dynamic whereby unassertive panellists may withhold or alter their input if they sense intimidation either real or imagined from other professional groups or strong personalities (49). Furthermore, the Delphi reduces participation bias as panellists are unfettered in their responses in the absence of others' scrutiny (49). Relevant materials, feedback reports and invitations to participate in the rounds were emailed to panellists individually per accepted practise (37, 51, 53).

### Design

Table 1 describes the two-phase Delphi study's design: the number and sequence of rounds, and the rounds specific to the intervention and the questionnaire. The interval between each round was set at 4 to 6 weeks.

**Table 1:** Delphi Panel design, timeline and response rates.

**Round**	**Date**	**Tasks Response Rates**	
One	May 2019	**Determination of OM health literacy scope and delivery**	
		Response: *n* = 30 out of 35 panellists (86%)	
		Health professionals *n* = 15 out of 20 (75%)	
		Education professionals *n* = 15 out of 15 (100%)	
		PHASE ONE: OM health literacy intervention	PHASE TWO: OM health literacy questionnaire
Two	September 2019	**Generation of lesson items and order** Response: *n* = 20 (57%) Health *n* = 11 (55%) Education *n* = 9 (60%)	**Clarification of type and order of questions** Response: *n* = 18 (51%) Health *n* = 9 (45%) Education *n* = 9 (60%)
Three	November 2019		**Consensus on questions** Response: *n* = 24 (69%) Health *n* = 16 (80%) Education *n* = 8 (53%)
	February 2020	**Individual review of selected lessons** Response: *n* = 10 (29%) Health *n* = 6 (30%) Education *n* = 4 (27%)	

The literature supports the use of Round One of a Delphi study to generate ideas and set the parameters around which the subsequent rounds are structured (37, 49–51, 53). This round used a questionnaire of simple and open-ended questions (51) to establish the target age, content ideas, essential OM health literacy skills, lesson settings, number and duration of lessons, and teaching strategies. Feedback to panellists is an essential component of Delphi (50). After Round One's results had been distributed to panellists, the study branched into two phases. The first phase had two rounds to inform the development of the intervention. The second phase had two rounds to inform the development of the questionnaire. The panellists were invited to consider the two phases of the intervention and questionnaire in parallel.

For the intervention phase, the second Delphi round focused on the content and teaching strategies for each lesson, asking “what topics need to be included?” and “how are they best taught?”. Panellists were also invited to make specific comments around content and teaching activities. In the final round, each lesson was reviewed by at least two relevant experts.

For the questionnaire phase, the second Delphi round provided a list of possible items and scales to test functional, interactive and critical OM health literacies. Panellists were invited to indicate the relevance of each question using a Likert scale, and to comment on language and question order. After applying their feedback, the third Delphi round sought final consensus.

### Data Collection and Analysis

Data was collected from four rounds using questionnaires on a Qualtrics® link as the web-survey software. This has been demonstrated as an efficient, cost-effective, convenient and user-friendly process to conduct Delphi studies (34) compared with questionnaires administered by electronic (38) or postal mail. Panellists responded to statements in these online questionnaires using Likert scales, rank ordering, open-ended questions and text boxes for optional comments (34, 54).

In accordance with accepted practise conventions, the acceptable level of consensus was set before the study began (53, 54) at 70% (33) in order to maintain rigour (34, 36, 50). The statistical analyses to assess consensus were calculating the mean, median (37, 50), frequency counts, ranking of Likert scale responses and rate of agreement using the formula [(strongly agree + somewhat agree) less (strongly disagree + somewhat disagree)] divided by [(strongly agree + somewhat agree) plus (strongly agree + somewhat agree) plus neither agree nor disagree] multiplied by one hundred per cent (53, 54). Qualitative responses were invited with open-field texts.

## Results

### Delphi Panel Composition

Of the 35 panellists, 20 were from health and 15 from education professions. Most panellists self-reported their areas of specialty, with some disclosing more than one (Table 2).

**Table 2:** Delphi panellists' occupations and specialties.

**Profession**	**Category**	**Main occupation**	**Disclosed specialties**
Health (*n* = 20)	Medicine (*n* = 4)	General Practitioner (*n =* 2)	Fertility (*n =* 2)
		Gynaecologist (*n =* 2)	Paediatric and adolescent (*n =* 1) Fertility (*n =* 1)
	Nursing (*n =* 2)	Registered nurse (*n =* 2)	Sexual health (*n =* 1) Fertility (*n =* 1) Midwife (*n =* 2)
	Allied healthcare (*n =* 8)	Accredited fertility awareness instructor (*n =* 3)	Billings LIFE™ (*n =* 2) Australian Council of Natural Family Planning Inc (*n =* 1) School teacher (*n =* 1) Women's health advocacy (*n =* 1)
		Nutritionist (*n =* 3)	Fertility (*n =* 2) Women's health advocacy (*n =* 1)
		Naturopath (*n =* 1)	Fertility (*n =* 1) Lactational consultant (*n =* 1)
		Psychologist (*n =* 1)	Chronobiology (*n =* 1)
	Public Health (*n =* 6)	Public Health practitioner (*n =* 1)	Registered nurse (*n =* 1)
		Women's health post-doctoral academic (*n =* 2)	Fertility (*n =* 1) Biomedicine (*n =* 1)
		Women's health advocate (*n =* 3)	School teacher (*n =* 1) Period poverty activist (*n =* 3)
Education (*n =* 15)	Curriculum (*n =* 4)	Consultant (*n =* 4)	School teacher (*n =* 2) Health & Physical Education (*n =* 1) Nutrition (*n =* 1)
	Teacher (*n =* 6)	Health & Physical Education (*n =* 4)	Relationships and Sexuality Education (*n =* 1) Well-being (*n =* 1)
		Science (*n =* 1)	Counsellor (*n =* 1)
		Technology (*n =* 1)	Personal development coach (*n =* 1)
	Counsellors (*n =* 5)	Psychologist (*n =* 2)	
		Pastoral carer (*n =* 3)	Teacher (*n =* 1) Chaplaincy (*n =* 2)

### Response Rates

Table 1 describes the time taken between rounds and the response rate per profession at each round. The overall response rate at Round One was 86%. At Round Two, it was 57% for the generation of lesson items and order, and 51% for clarification of the type and order of questions. At Round Three, the response rate for the individual review of selected lessons was 29% and 69% for the consensus on questions.

### Round One: Determination of OM Health Literacy Scope and Delivery

The agreed mean age at which the intervention is to be targeted was 13.39 years (range 12 to 16 years). There was little difference between the mean age recommended by the health panellists (13.25 years) compared to the education panellists (13.57 years).

Based on an accumulation of ranking preference whereby the score of “1” indicated each panellist's highest preference, the panellists selected items to be emphasised in the proposed intervention and questionnaire. Their top five preferences for overall coverage of OM health education were typical OM cycle parameters (score 74), physiology (score 139), cultural beliefs (score 184), whole person approach (score 199), and body image (score 201).

Regarding OM cycle dysfunctions, the panellists' top five ranked preferences were dysmenorrhoea (score 54), premenstrual syndrome (score 91), irregular cycles (score 111), atypical bleeding patterns (score 123) and endometriosis (score 159).

For OM health literacy skills, the top five ranked preferences were observational skills (score 90), skills to record and interpret OM cycle symptoms (score 112), self-expression skills (score 154), communication skills with healthcare providers (score 156) and skills to critique OM health information (score 181).

In terms of settings for teaching, the five most frequent selections were school health lessons (score 28), well-being lessons (score 25), a medically based setting such as a General Practitioner clinic (score 13), school science lessons (score 10), and community-based settings such as social (score 10) or sports clubs (score 9).

For teaching and learning strategies, the five most frequent selections were small group discussions (score 27), students charting their own OM cycles (score 26), PowerPoint slides (score 22), visual media (score 19), and demonstration aides (score 18).

In terms of the number of 45–50-min sessions needed to teach OM health literacy skills, the median was five sessions (range two to 24). The health panellists recommended a median of eight sessions. Four health panellists recommended that these are spread over 3 to 6 months. One gynaecologist explained a “12-week minimum to “own” their fertility, plus 3 months more to explore the implication on their lives.” In contrast, the education panellists recommended a mean of five sessions, with three recommending “a minimum of two sessions biannually from Year 5 to Year 11 building on competency.” The additional comments from two health panellists concurred with “the intervention should be introduced as young as 10 years,” with a “follow up over several years.”

Two education and four health panellists highlighted challenging the secrecy, shame and stigma of menstruation. One teacher advised that “many of the 13–16-year-old girls I have taught find it very difficult to talk about their own menstrual cycle—it's embarrassing!” Four education and one health panellists suggested that education is better delivered by female teachers in a same sex setting.

Fourteen panellists suggested external contributors. Six education and four health panellists recommended family inclusion. Eight education and two health panellists recommended “outside voices” as guest speakers, which may include healthcare professionals and “female role models.” Three education and one health panellists suggested the inclusion of older girls for peer-based teaching.

### Phase One Intervention: Round Two—Generation of Lesson Items and Order

The findings of Round One informed the creation of a draft intervention designed for girls (Table 3) (53). Reconciliation of the time and session differences between the health and education panellists had been achieved by spacing nine lessons over 12 weeks, and locating three lessons outside curriculum time. This simultaneously satisfied the panellists' recommendation to include families. Twenty panellists commented on the draft intervention (Table 1). Table 3 describes the consensus achieved for each lesson, giving an overall consensus of 82%. In general, panellists recommended that “students need to start feeling safe” and “as they become more confident, strategies can become more open” with class discussions and guest speakers.

**Table 3:** Round Two consensus of the draft OM health literacy intervention.

**Lesson and Location**	**Content**	Health literacy*	**Consensus†**	**Concerns raised by panellists**
1 Home-based	Genealogy OM cycle as a personal health monitor	F I	85%	Discomfort in discussing OM health for parents (*n =* 9) and for daughters (*n =* 4)
2 School- family event	Rite of passage Cultural beliefs	I C	89%	Low parent attendance due to time constraints (*n =* 8)
3 In class	Typical OM cycle overview	F	83%	Sufficient time required (*n =* 2)
4 In class	Charting skills	F I	79%	Confusion in personally applying lesson 3 (*n =* 7), requiring regular private reviewing of charts (*n =* 3)
5 In class	Common OM dysfunctions	F I	89%	Risk of provoking anxiety (*n =* 2)
6 Home-based	Critique of misinformation	C	89%	Unlikely to be completed (*n =* 7)
7 In class	Menstrual stigma	I C	72%	Girls' discomfort (*n =* 3)
8 In class	Lifestyle and remedies for OM dysfunctions	F I	78%	Requirement to present evidence-based remedies only (*n =* 1)
9 In class	Communication skills (with healthcare professionals)	I	72%	Caution in selecting qualified speakers (*n =* 3) and students' discomfort with strangers (*n =* 4)

* *F, Functional; I, Interactive; C, Critical Based on the Health Outcome Model (28).*

† *The formula used to calculate consensus is the Rate of Agreement, specifically: [(strongly agree + somewhat agree) less (strongly disagree + somewhat disagree)] divided by [(strongly agree + somewhat agree) plus (strongly agree + somewhat agree) plus neither agree nor disagree] multiplied by 100%*.

The home-based Lesson 1 included a guided discussion of family history such as genealogy and an appreciation of the cycle as a vital sign (55) to monitor personal health. The possible reasons for discomfort with this lesson included daughters' reluctance to discuss OM with their fathers, cultural prohibitions and mothers' poor OM experiences.

Panellists supported a rite of passage school-event for Lesson 2 as “an opportunity for students to discuss with their mothers about her experiences, even a grandmother's experiences would be valuable.” Two panellists suggested multiple sessions or flexible scheduling to overcome possible low attendance.

Understanding the function and typical parameters of the OM cycle had been ranked as the most important content in Round One. This formed an overview of a typical OM cycle for the third in-class lesson. Panellists cautioned time sufficiency and “having the right materials pitched at the right level for their age.”

The fourth in-class lesson aimed to teach girls' how to apply Lesson 3 to their personal OM experiences. To counteract possible confusion of achieving this, one gynaecologist recommended establishing luteal length to validate ovulation. This presupposed existing support of regular individual reviewing of girls' charts to ensure their consistency in observations, recording and interpreting of their OM biomarkers.

The fifth lesson focused on common OM dysfunctions. Seven panellists recommended sensitive handling to manage “fear/concern” and “anxiety” in the event of “students overreacting” and having a “reliance on ‘Dr Google'.” Suggestions included “a safe environment and appropriate channels for girls to get support/talk to someone” and to “repeat discussions even if they are off your timetable.”

Although panellists strongly supported the sixth home-based lesson to critique OM health misinformation, it was indicated that it would likely not be completed unless it were an assessed task.

The seventh in-class lesson included group work activities aimed at exploring potential shame and stigma surrounding the OM cycle. This is reflected in the comment, “discomfort may be one challenge, but I think that's expected and it's the whole point of having this lesson.”

The impact of lifestyle and remedies for common OM dysfunctions were to be covered in the eighth lesson. Overall, the panellists supported “holistic health,” but it was indicated that remedies may be “fraught with difficulties about evidence-based solutions.”

The final lesson invited guest speakers to role-play communication strategies. A robust process to select and inform guest speakers was recommended, particularly given the possible discomfort of students around this content.

### Phase One Intervention: Round Three—Individual Review of Selected Lessons

Since the 82% consensus had surpassed the pre-determined 70% consensus, the first phase was thus completed. Based on Round Two feedback, the final version of the intervention was drafted. Lessons which were specific to panellists' occupations were forwarded for their review. Ten panellists responded in this Round (Table 1).

Detailed materials for the first, second, sixth, seventh and ninth lessons were distributed to education panellists according to their specialties. Health panellists reviewed the scientific accuracy of materials for the third, fourth, fifth and eighth lessons. The medical panellists recommended that the emphasis should be on teaching students to accurately report their OM symptoms and that neither teachers nor students attempt diagnoses of OM dysfunctions.

Each lesson was independently reviewed by at least two panellists. Their recommendations finalised the drafting of the OM health literacy intervention. Face validity was subsequently conducted with adolescent girls, their parents, teachers and school healthcare professionals (33) and will be reported separately.

### Phase Two Questionnaire: Round Two—Clarification of Type and Order of Questions

Based on the findings in Round One and using previously validated questions where possible (45–48), a draft OM health literacy questionnaire was developed (53). Panellists were invited to indicate the relevance of 141 questions using a Likert scale from “Highly relevant” to “Not relevant,” including 42 invitations for detailed suggestions to indicate if core questions were missing with free text answers. Eighteen panellists commented on the questionnaire (Table 1). For functional health literacy, 50 questions focused on finding and understanding information, including factual knowledge of reproductive physiology, menses, ovulation, cycle normality, charting conventions and fertility. The rate of agreement reached 25%. For interactive health literacy, 33 questions centred around social skills including attitudes towards the cycle and communication with healthcare providers. The rate of agreement reached 20%. A section for postmenarcheal girls included 44 questions about personal cycle tracking, experiences of common OM dysfunctions and OM health goal setting. The rate of agreement reached 26%. For critical health literacy, 14 questions centred on social awareness of the OM cycle and included problem-solving challenges to test information appraisal. The rate of agreement reached 26%. The overall rate of agreement of 24% failed to reach the pre-determined 70% consensus. After providing feedback based on panellists' suggestions of additions, deletions, rewording, and adjusting the order of items, the next round commenced.

### Phase Two Questionnaire: Round Three—Consensus on Questions

The findings from Round Two were used to revise the draft questionnaire. A total of 53 questions were created. Panellists were invited to indicate the relevance of each question using a Likert scale from “Highly relevant” to “Not relevant.” Opportunity to add comment was provided in a text box for each question (34). Twenty-four panellists responded in this Round (Table 1).

Table 4 presents the overall consensus achieved across all 53 questions as 84%, with a range of 71% to 96%. This was divided into the Model's three domains for functional, interactive and critical health literacies (28), which were then divided into sub-categories.

**Table 4:** Round Three consensus of the draft OM health literacy questionnaire.

**Health literacy domain**	**Categories within each Domain**	**Number of questions**	Overall % consensus*	**Range of consensus**	
				**Highest %**	**Lowest %**
Functional		**13**	**88%**	**92%**	**75%**
	Finding information	3	81%	92%	75%
	Comprehension	10	91%	92%	88%
Interactive		**22**	**82%**	**92%**	**71%**
	Personally apply information	10	81%	96%	75%
	Communication skills	8	81%	83%	71%
	Setting goals	4	85%	92%	75%
Critical		**18**	**83%**	**96%**	**71%**
	Analysis of cycle problems	3	86%	88%	83%
	Social awareness	15	83%	92%	71%
**Total of health literacy domains**		**53**	**84%**	**96%**	**71%**

* *The formula used to calculate consensus is the Rate of Agreement, specifically: [(highly relevant + quite relevant) less (not relevant)] divided by [(highly relevant + quite relevant) plus (not relevant) plus indifferent] multiplied by 100%. The bold values report on the 3 domains in total of column one (functional, interactive, critical). The light values report on the categories within these domains (column two)*.

Comments from four panellists for functional health literacy emphasised the need for the intervention to clearly teach the meanings of the terms used in describing the OM cycle. For example, a health panellist reflected that “I've had adults get the terms ‘cycle' and ‘period' mixed up.” Three health panellists recommended additional items on gamete survival times and the finite number of oocytes.

For interactive health literacy, health panellists suggested an additional item for goal setting such as “I focus on managing my energy levels at different times of my cycle.” Two health panellists suggested additional items concerning what might have already been done to address a pre-existing OM disturbance, and the effectiveness of self-remedies.

For critical health literacy, comments from education panellists centred on additional items regarding the selection of resources, such as “do you cross check with other sources to make sure that the information is correct?”. An education panellist recommended that questions be oriented to healthy living rather than fertility as “teenagers are not worried about any inability to have a baby at this stage.” Finally, regarding social awareness, one health panellist suggested an additional item that “the first period is an important milestone that means a girl is becoming a woman.”

## Discussion

This research accommodated the cross-curriculum priorities of the Australian curriculum (56) because it spanned both HPE and Science. The challenges in achieving consensus will be discussed by exploring the conduct of the Delphi study, the areas of concordance and the resolution of discordance.

### Conduct of the Online Two-Phase Modified Delphi Study

Although this Delphi study appears weighted towards the health profession, heterogeneity (23, 52) was preserved with the presence of two school-teachers and five women's health advocates as disclosed specialties (Table 1). The overall composition of the Delphi panellists therefore indicates a balance of expertise between and among health and education professionals. This is notwithstanding that where expertise is imperfect, the wisdom-of-crowds literature supports the validity of group consensus judgements (36, 52).

There was vigilant preservation of anonymity (21, 50, 51). Additionally, individual communication (53) may have contributed to panellists' independence of ideas and reflections.

This study's time frame gave the panellists flexibility to respond according to their own schedules. Incorporating the opt-in opt-out model (49) did not exclude panellists from responding in later Rounds if they had not responded in an earlier Round. An increase in the response rate in Round Three of the Questionnaire (Table 1) gives suggestive support for this.

This two-phase modified study achieved an ambitious goal of informing both an intervention and questionnaire. Round One commenced as a classic Delphi which gave panellists freedom to generate ideas (37, 49–51, 53). Creating two Delphi studies through two concurrent phases offered time and resource efficiencies in assembling a panel and conducting the study. Furthermore, it maintained the advantages of the classic Delphi methodology through the structured framework within both phases which efficiently funnelled feedback and iterative revisions (37, 54). This allowed the panellists to hold the overall context of the study (51). For example, in Round Three of the intervention, panellists reviewed lessons within their specialty whilst knowing that relevant tangents were being reviewed by the other suitably qualified panellists. To our knowledge, this research contributes to the flexibility for which Delphi studies are renowned (49, 53).

There is merit in incorporating school students' perspectives on OM cycle education. Having established content validation of the draft intervention and questionnaire through the Delphi Panel (36, 54), the next step in the study's protocol is face validation (33). This aims to capture not only the perspectives of school students, but also those of teachers and school healthcare professionals. After face validation, the questionnaire will undergo test-retest for reliability, and will be used in the pre- and post-testing of the intervention's trial.

### Areas of Concordance

In Western Australia, the average age for menarche is 12.7 years (3). The panellists agreed that the mean age at which to aim the intervention is older than this, which aligns with the onset of ovulatory cycles from 1 to 3 years postmenarche (57).

The panellists recommended a whole person approach rather than an exclusive focus on biology (58). However, comprehension of biology needed to be sufficient for an individual girl to understand how her own body works, and to effectively communicate with healthcare providers. Couching biology within the social and emotional aspects of the OM cycle touched on body image and mental health. The panellists sought redress of the shame, secrecy and stigma surrounding menstruation (12, 13). A strengths-based approach (59) emphasised the cycle as a vital sign (55) of good health in its own right, and allowed alternative positive discourses of menstruation (60, 61) to be opened. This approach contrasts with the current linking of menstruation and pregnancy (62) in the HPE curriculum and with the deficit-orientation (63) of menstrual health programmes (11) which solve (59) OM dysfunctions.

Nonetheless, the panellists recognised the negative impact of OM dysfunctions on health, including iron deficiency (4, 64–66). One medical panellist also recommended a focus on “acne, weight and hair changes.” Furthermore, the OM cycle and / or its dysfunctions are relevant to inter alia asthma, epilepsy, migraines (67) and mental health (68), including anxiety (67), eating disorders (67, 69–71), negative body image (69, 72–75), and non-suicidal self-injury (76, 77). The whole person approach captured both biological and mental health experiences of the OM cycle.

This approach contrasts with the tendency of most menstrual health programmes to be based on a single issue (11). Interestingly, the panellists ranked endometriosis below premenstrual syndrome, irregular cycles and atypical bleeding patterns. Endometriosis affects 11% of the Australian female population (78). Panellists' recommendations point to accommodating endometriosis within dysmenorrhoea rather than as a dedicated subject. The medical panellists recommended that girls are not to self-diagnose, but rather develop accurate reporting skills of cycle symptoms. Albeit for a different reason, the education panellists also concluded that it is inadvisable to cause adolescent girls' anxiety for an OM dysfunction which may not affect 89% of them. A similar approach was adopted for Polycystic Ovarian Syndrome, which affects up to 21% of the population (79) and can be challenging to diagnose in adolescents (80).

Whilst adolescents are dependents at home, schools can also address their needs for health education and health promotion to support their natural development (10). The World Health Organization's Health Promoting Schools (HPS) framework recognises this as a whole-school approach (81). It proposes an interrelation between schools' formal curriculum, ethos, physical environment and engagement with family and the external community (10). Therefore, the intervention was mapped to the formal HPE and Science curricula; included the active participation of the school healthcare professionals; orchestrated student-family dialogues; and allowed for the inclusion of the community in promoting OM health. This allowed for the possibility of expanding the “collective health literacy” skills of key individuals such as parents, teachers and school healthcare professionals (82).

### Resolution of Discordance

The HPS framework encourages engagement with external healthcare professionals to support the school's formal health education and promotion (10), and this was supported by the education panellists (22, 58). However, it was not as keenly supported by the health panellists. Possibly, the extra demands to present in schools in addition to managing busy health practises were dissuasive. One possible resolution would be for schools to invite healthcare professionals from within the school's parent-body. This may help overcome known challenges in schools engaging family and community (10).

There was also disagreement between health and education panellists over the time for girls to learn their cycles. The intervention resolved this in two ways.

Firstly, learning is extended vertically through the school grades. The intervention recognised the prior learning of early years in preparation for menarche, and extended teaching to recognising ovulation as a sign of good health (83). Around Grades 8 to 9, girls' typical growth trajectory means their cycles are becoming ovulatory (57, 84). This progressive development of girls is consistent with current pedagogy that health programmes align with their cumulative knowledge and experience developed over pubescence. The intervention is therefore developmentally appropriate in shifting focus from menstruation's obvious bleeding to the hidden event (85) of ovulation which governs menstruation itself (83, 86).

Secondly, as part of the HPS framework, the intervention included the school's healthcare team in formal OM health lessons. Since ovaries function irrespective of school grade, support would be consistently available even in the absence of formal lessons, which is particularly comforting for those with OM dysfunction.

## Conclusion

This two-phase modified Delphi study built consensus of over 80% between two distinct professions of education and health. Its anonymity facilitated by the online setting helped avoid undue influence amongst panellists and allowed their independent contribution (21, 50). The use of Qualtrics® as the online survey facilitated the speed of survey administration, data collection and feedback collation at each Round (34). However, the reproducibility of this study may be reduced because of panellists' potential fatigue (49). The Delphi methodology is useful for content validation (36, 54). This study informed the development of a scientifically accurate and pedagogically appropriate intervention for adolescents to acquire OM health literacy and a questionnaire to assess this. As determined by a prior protocol, these school resources were prepared for final validation in advance of their trial (33).

## Data Availability Statement

The datasets presented in this article are not readily available because Ethics restricted the sharing of data. Requests to access the datasets should be directed to Felicity.Roux@postgrad.curtin.edu.au.

## Ethics Statement

The studies involving human participants were reviewed and approved by the Human Research Ethics Committee at Curtin University (HREC 2018-0101-02) and Catholic Education Western Australia (RP2018/44). The patients/participants provided their written informed consent to participate in this study.

## Author Contributions

FR, SB, HC, and JH contributed to the conception and design of the study. FR collected, analysed, reported data, and wrote the manuscript. SB, HC, and JH reviewed data analyses and feedback reports and reviewed the manuscript. All authors contributed to the article and approved the submitted version.

## Funding

This work was supported by the Australian Government Research Training Program Scholarship under Grant CHESSN8617438119 and by Curtin Medical School's 2020 Seed Grant for Trials.

## Conflict of Interest

The authors declare that the research was conducted in the absence of any commercial or financial relationships that could be construed as a potential conflict of interest.

## Publisher's Note

All claims expressed in this article are solely those of the authors and do not necessarily represent those of their affiliated organizations, or those of the publisher, the editors and the reviewers. Any product that may be evaluated in this article, or claim that may be made by its manufacturer, is not guaranteed or endorsed by the publisher.
